# Persistent bilateral pneumothorax after robotic-assisted inguinal hernia repair: possible relevance to recent esophageal cancer surgery — a case report

**DOI:** 10.1186/s40981-023-00630-y

**Published:** 2023-06-22

**Authors:** Seiji Ishikawa, Kaori Shirakawa, Yui Kuroda, Yukinori Yube, Shinji Mine, Masakazu Hayashida, Izumi Kawagoe

**Affiliations:** 1 Department of Anesthesiology and Pain Medicine, Faculty of Medicine, Juntendo University, 2-1-1, Hongo, Bunkyo-Ku, Tokyo, 113-8421 Japan; 2 Clinical Training Center, Juntendo University Hospital, 3-1-3, Hongo, Bunkyo-Ku, Tokyo, 113-8431 Japan; 3 Department of Esophageal and Gastroenterological Surgery, Faculty of Medicine, Juntendo University, 2-1-1, Hongo, Bunkyo-Ku, Tokyo, 113-8421 Japan

**Keywords:** Pneumothorax, Robot-assisted inguinal hernia surgery, Esophagectomy, Postoperative complication, Case report

## Abstract

**Background:**

Postoperative pneumothorax is a well-known but relatively rare complication after laparoscopic surgery. There has been no report describing pneumothorax that persisted for a week or more after laparoscopic surgery. Herein, we report a case of bilateral pneumothorax after laparoscopic surgery, which appears to have occurred by a different mechanism than previously described.

**Case presentation:**

A 65-year-old male, with a past history of esophagectomy and retrosternal gastric tube reconstruction 4 months earlier, underwent a robotic-assisted inguinal hernia repair. Postoperative chest x-rays revealed the development of bilateral pneumothorax, which became worse on postoperative day (POD) 1 and took more than 9 days to resolve spontaneously. We assumed that intra-abdominal gas replaced by the air after pneumoperitoneum might have migrated into thoracic cavities through an opened esophageal hiatus or along the retrosternal route.

**Conclusions:**

Laparoscopic surgery after radical esophagectomy may be associated with an increased risk of postoperative pneumothorax.

## Background

Postoperative pneumothorax is a well-known but relatively rare complication after laparoscopic surgery. Studies of laparoscopic surgery as a whole have reported a frequency of postoperative pneumothorax ranging from 0.01 to 1.9% [1–3]. In most cases, pneumothorax related to laparoscopic surgery, including that for an inguinal hernia repair, develops in a unilateral chest cavity during or soon after surgery and disappears quickly without the need for interventions [4, 5].

Herein, we report a case of persistent, bilateral pneumothorax after a robotic-assisted inguinal hernia repair, which became evident not on postoperative day (POD) 0 but on POD 1 and took more than 9 days to resolve spontaneously. The patient had undergone esophageal cancer surgery 4 months earlier, which could have contributed to the occurrence of this complication.

This article was prepared and written in accordance with the CAse REport guidelines. Written informed consent was obtained from the patient for publication of this case report and accompanying images. The Institutional Review Board of Juntendo University Hospital approved publication of this case report (approved number, JHS22-027, November 17, 2022).

## Case presentation

**Fig. 1:**
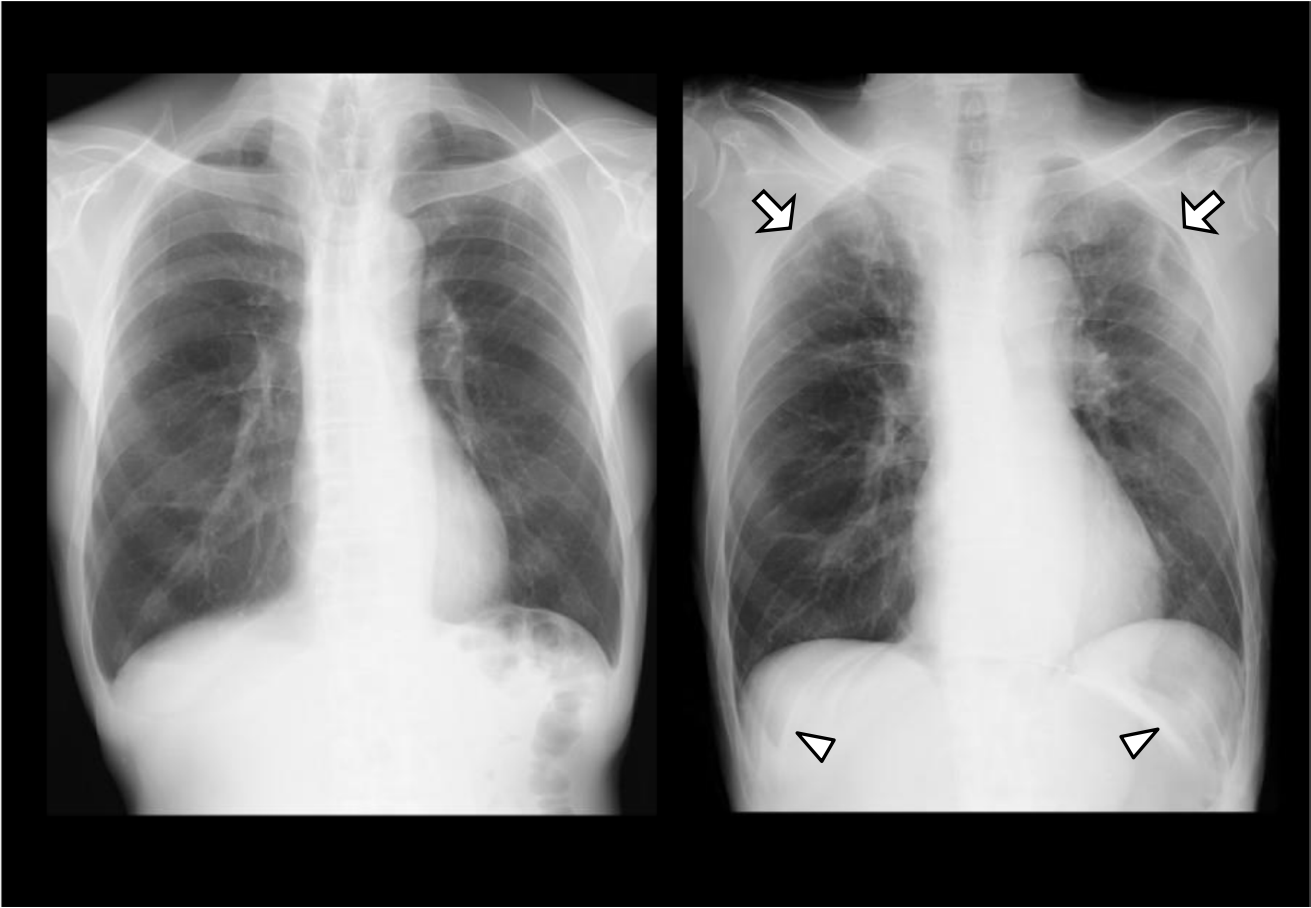
Left: A chest radiograph in the upright position before surgery. Right: A chest radiograph in the supine position on postoperative day 0 showing slight bilateral pneumothorax (arrows) and the deep sulcus sign (arrowheads)

A 65-year-old male, 175 cm, 55 kg, was scheduled for a robotic-assisted repair for a left inguinal hernia. The patient had a history of medically controlled cervical spondylosis and past coronavirus-2 (SARS-CoV-2) infection. In addition, he had undergone a right inguinal hernia repair under general anesthesia 10 years before and a thoracoscopic subtotal esophagectomy and retrosternal gastric tube reconstruction for esophageal cancer 4 months before. Preoperative examinations, including blood and urine tests, an electrocardiogram, chest and abdominal radiographs, and spirometry (vital capacity as percent predicted, 104.6%; forced expiratory volume in 1 s as percent of forced vital capacity, 77.0%), revealed no abnormality except for a low hemoglobin level (11.4 g/dL). A chest radiograph showed no pneumothorax (Fig. 1 Left). Furthermore, a computed tomography (CT) taken before the esophagectomy had shown no emphysematous lung cysts.

Anesthesia was induced with propofol, remifentanil, and rocuronium and maintained with desflurane and remifentanil after tracheal intubation. Bilateral rectus sheath blocks with levobupivacaine were performed under ultrasound guidance prior to surgery. During surgery, the lungs were ventilated with a volume-guarantee pressure-controlled mode employing a fraction of inspired oxygen, 50%; tidal volume, 450–500 mL; positive end-expiratory pressure, 5 cmH_2_O; and respiratory rate, 10–14 per minute.

With the patient placed in the supine position, 8-mm ports were inserted via skin incisions at the umbilical and right as well as left lateral abdominal walls for abdominal carbon dioxide insufflation at a pressure of 10 cmH_2_O and subsequent laparoscopic procedures. Then, a robotic-assisted inguinal hernia repair using the da Vinci Xi Surgical System (Intuitive Surgical, Sunnyvale, CA, USA) was performed, while the patient was placed in a 10-degree head-down position. The peritoneum around the hernia orifice was dissected widely, a hernia mesh placed over the dissected area was fixed on fasciae and ligaments, and the peritoneum over the mesh was closed. No surgical manipulation of the upper abdominal organs or the diaphragm was performed. Intraoperatively, percutaneous arterial oxygen saturation (SpO_2_) and end-tidal carbon dioxide partial pressure remained at 99–100% and 35–40 mmHg, respectively. The peak airway pressure during the Trendelenburg position remained around 20–25 cmH_2_O. Fentanyl 200 μg and acetaminophen 1000 mg were intravenously administered for immediate postoperative analgesia. Durations of surgery and anesthesia were 82 and 143 min, respectively. No subcutaneous emphysema was noted.

Emergence from anesthesia was rapid, and the extubation was uneventful. In the postanesthesia care unit, SpO_2_ remained between 99 and 100% with supplemental oxygen delivered at 4 L/min via a mask. The patient complained of no pain or dyspnea and returned to the ward after a 30-min stay.

**Fig. 2:**
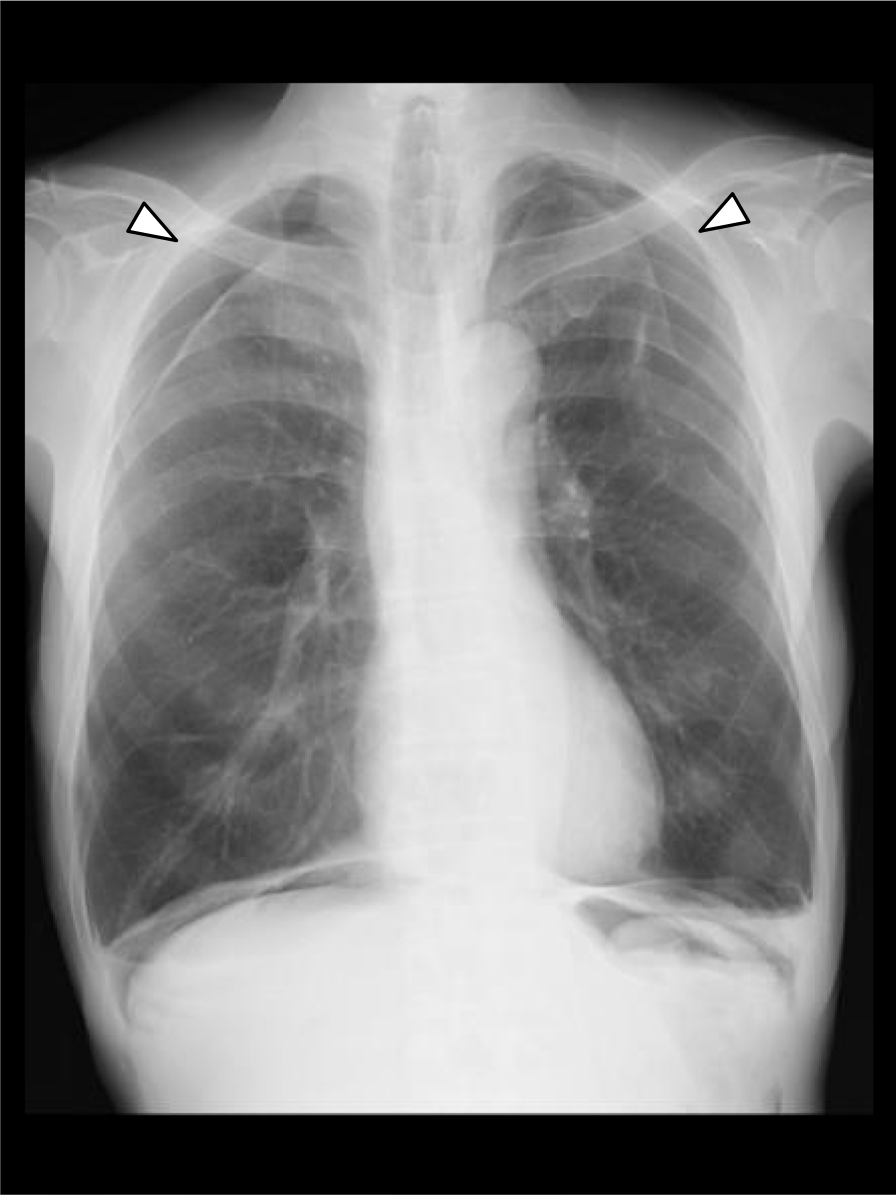
A chest radiograph in the upright position on postoperative day 1 showing evident bilateral pneumothorax (arrows)

**Fig. 3:**
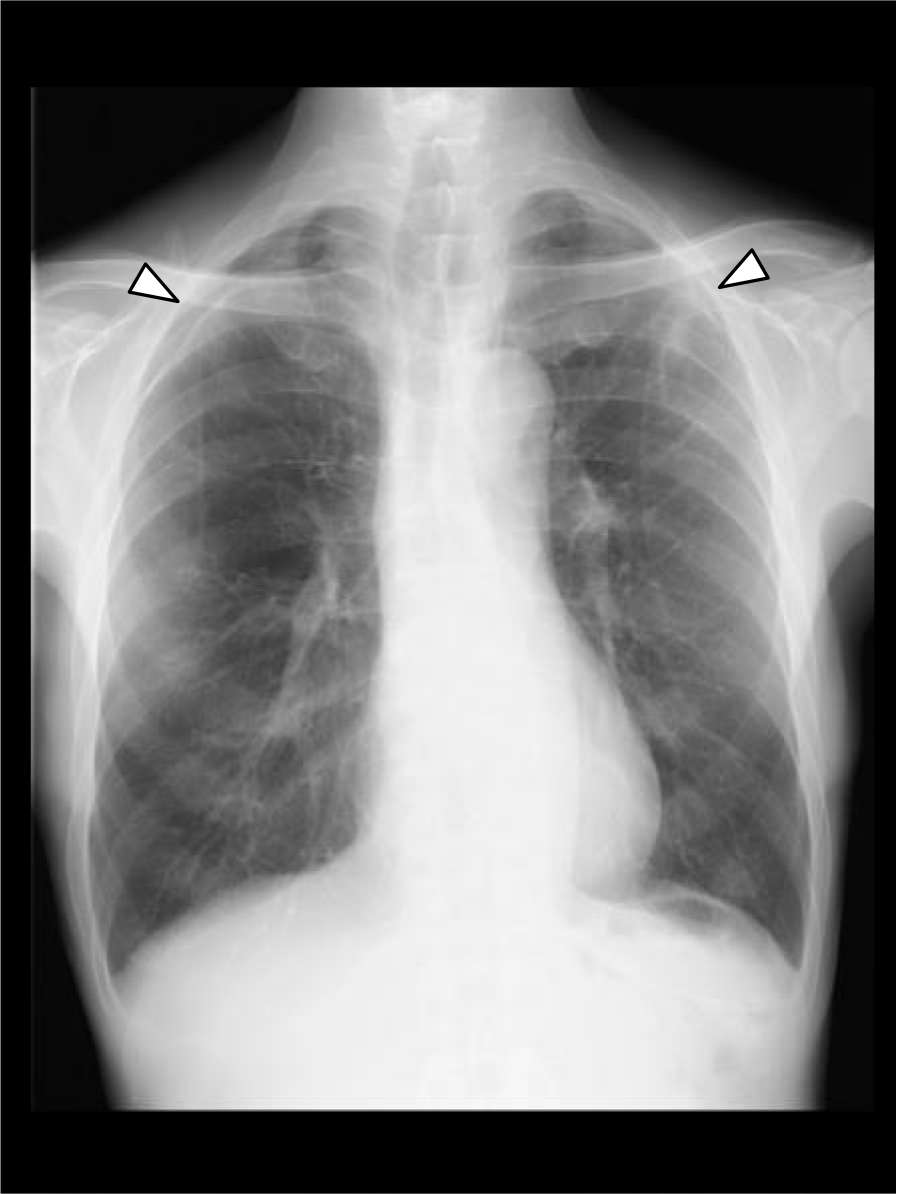
A chest radiograph in the upright position on postoperative day 9 showing slight residual bilateral pneumothorax (arrows)

In the ward, supplemental oxygen was continued for 3 h. Even after its termination, SpO_2_ remained at 98–99%. A supine anteroposterior chest radiograph, taken approximately 2 h after surgery, showed only slight bilateral pneumothorax and the deep sulcus sign, which represents lucency of the lateral costophrenic angle extending toward the hypochondrium and suggests the presence of a pneumothorax in supine position [6] (Fig. 1 Right). On POD 1, SpO_2_ remained at 97–98% without supplemental oxygen. However, a follow-up upright posteroanterior chest radiograph showed evident bilateral pneumothorax (Fig. 2). Because of no symptoms of dyspnea or chest pain, the patient was to be followed up without specific interventions. The patient was discharged from hospital on POD 3, since a chest radiograph on POD 2 showed no worsening of pneumothorax. At the outpatient visit on POD 9, a chest radiograph showed slight residual bilateral pneumothorax (Fig. 3). A chest CT taken on POD 44 showed no remaining pneumothorax.

## Discussion

In this case, postoperative pneumothorax occurred bilaterally, deteriorated from POD 0 to POD 1, and persisted for more than 9 days. These characteristics are quite different from those reported previously. In most cases of pneumothorax related to laparoscopic surgery, unilateral pneumothorax develops during or soon after surgery and disappears quickly without interventions [4, 5]. To our knowledge, there has been only one report describing pneumothorax that became evident the day after laparoscopic surgery [7], and there have been no reports describing pneumothorax that persisted for a week or more after laparoscopic surgery.

Several mechanisms for the development of pneumothorax after laparoscopic surgery have been reported, including iatrogenic injury on the diaphragm [8], a rupture of an emphysematous lung cyst, and carbon dioxide migration from mediastinal and/or subcutaneous emphysema into the thoracic cavity [9]. However, these mechanisms did not seem applicable to our case because our patient underwent lower abdominal surgery without any surgical manipulation in the upper abdominal cavity, had no emphysematous lung cysts on a previous chest CT, and had no mediastinal or subcutaneous emphysema on physical or radiological examinations. Furthermore, although pneumoperitoneal gas can migrate from the abdominal cavity into the thoracic cavity via originally existing channels, such as the thoracoabdominal hiatus (Bochdalek foramen), aortic hiatus, foramen Morgani, or a congenital defect of the diaphragm [10], such mechanisms did not seem applicable to our case either, since in such situations, carbon dioxide pneumothorax becomes most evident immediately after surgery and rapidly resolves afterwards reflecting rapid carbon dioxide absorption. In contrast, our case exhibited bilateral pneumothorax that became more evident on POD 1 than POD 0 and persisted for more than 9 days.

The mechanism of the pneumothorax development in this case is not of a provable nature. Although it is difficult to draw definite conclusions, the progression and resolution of bilateral pneumothorax specific to our case might be explained by the following mechanisms. First, the air may have replaced intra-abdominal gas after completion of pneumoperitoneum, while the surgeons were lifting the abdominal wall during wound closure to prevent abdominal organs being entrapped. Therefore, the air, and not carbon dioxide, may have resulted in the subsequent long-lasting pneumothorax. Second, we assumed that the air migrated into thoracic cavities through an incompletely closed esophageal hiatus or along the retrosternal route associated with the previous esophagectomy. Third, the development of evident pneumothorax on POD 1, and not on POD 0, may have resulted from the upright position that the patient took during the process of postoperative ambulation, which should facilitate the shift of the remaining intra-abdominal gas into thoracic cavities. Thus, pneumothorax may have developed due to migration of the air rather than carbon dioxide and after pneumoperitoneum rather than during pneumoperitoneum. It should be noted that intra-abdominal gas after pneumoperitoneum disappears within 24 h in more than half of patients, but it can continue to remain in situ for more than a week in some patients [11]. Since esophageal cancer surgery involving right-sided thoracoscopic procedures can result in an incidental injury to the left parietal pleura, the bilateral pneumothorax might develop due to the communication between the two chest cavities [12, 13], although its presence was not evidently proven in this case.

It is difficult to exactly indicate the onset time of pneumothorax in our case. However, it seemed plausible that pneumothorax occurred at a certain time after pneumoperitoneum was ended and before the first postoperative chest radiograph was taken approximately 2 h after surgery, as the gas entrapped in thoracic cavities was more likely to be the air than carbon dioxide, and pneumothorax, albeit slight, was detected with this first radiograph.

We experienced a case of persistent bilateral pneumothorax that developed the day after a robotic-assisted inguinal hernia surgery. Although the mechanism of the pneumothorax was not clear, it might be related to previous esophageal cancer surgery. Our experience suggests that laparoscopic surgery after esophageal cancer surgery, at least performed recently, can be associated with an increased risk of pneumothorax as a postoperative complication.

## Data Availability

Data sharing is not applicable to this article as no datasets were generated or analyzed during the current study.

## Abbreviations

POD: Postoperative day
SARS-CoV-2: Coronavirus-2
CT: Computed tomography
SpO_2_: Percutaneous arterial oxygen saturation
